# Effect of the Interaction Between Pre-pregnancy Body Mass Index and Fresh/Frozen Embryo Transfer on Perinatal Outcomes of Assisted Reproductive Technology-Conceived Singletons: A Retrospective Cohort Study

**DOI:** 10.3389/fendo.2020.560103

**Published:** 2020-09-25

**Authors:** Pengfei Qu, Yang Mi, Doudou Zhao, Min Wang, Shaonong Dang, Wenhao Shi, Juanzi Shi

**Affiliations:** ^1^Translational Medicine Center, Northwest Women's and Children's Hospital, Xi'an, China; ^2^Departments of Pediatrics and Neonatology, Children's Hospital of Fudan University, Shanghai, China; ^3^Department of Obstetrics, Northwest Women's and Children's Hospital, Xi'an, China; ^4^Assisted Reproduction Center, Northwest Women's and Children's Hospital, Xi'an, China; ^5^Department of Epidemiology and Health Statistics, School of Public Health, Xi'an Jiaotong University Health Science Center, Xi'an, China

**Keywords:** overweight, assisted reproductive technology, perinatal outcomes, interaction, cohort study

## Abstract

**Objective:** To demonstrate the association between pre-pregnancy maternal overweight, obesity, and perinatal outcomes of singletons conceived by assisted reproductive technology (ART).

**Design:** Retrospective cohort study from 2006 to 2015 data from a single ART center.

**Setting:** Assisted Reproduction Center, Northwest Women's and Children's Hospital, Xi'an, Northwestern China.

**Patients:** We included 7,818 women undergoing ART and their singleton infants.

**Interventions:** None.

**Main Outcome Measure:** The primary outcome measures were preterm birth (PTB), macrosomia, low birth weight, small for gestational age, and large for gestational age (LGA).

**Results:** We experienced an increase in the risk of PTB, macrosomia, and LGA in overweight and obese groups compared with that in normal-weight groups [PTB: overweight vs. normal weight: odds ratio [OR] = 1.44, 95% CI: 1.18–1.75; obesity vs. normal weight: OR = 1.53, 95% CI: 1.04–2.25; macrosomia: overweight vs. normal weight: OR = 1.78, 95% CI: 1.48–2.14; obesity vs. normal weight: OR = 2.16, 95% CI: 1.52–3.06; LGA: overweight vs. normal weight: OR = 1.63, 95% CI: 1.39–1.90; obesity vs. normal weight: OR = 2.11, 95% CI: 1.57–2.83]. We observed a significant interaction between maternal BMI and fresh/frozen embryo transfer on PTB and LGA (*P* = 0.030; *P* = 0.030). Fresh embryo transfer significantly increased the effect of maternal BMI on LGA (fresh: OR = 1.14, 95% CI: 1.10–1.18; frozen: OR = 1.09, 95% CI: 1.04–1.13), and frozen embryo transfer increased the effect of maternal BMI on PTB (fresh: OR = 1.03, 95% CI: 0.99–1.08; frozen: OR = 1.09, 95% CI: 1.04–1.15).

**Conclusions:** Pre-pregnancy maternal overweight and obesity were associated with higher risks of PTB, macrosomia, and LGA in ART-conceived singletons. These associations were affected by the timing of embryo transfer (fresh/frozen embryo transfer). Therefore, we recommend women before ART to maintain a normal BMI for the prevention of adverse perinatal outcomes.

## Introduction

In recent decades, there has been an alarming rise in the incidence of overweight and obesity worldwide (1, 2). China being the largest cannot be left out as economic development, and westernization is gradually gaining new grounds leading to a sharp rise in prevalence (3, 4). The Report on Chinese Resident Nutrition and Chronic Diseases published in 2015 revealed that the prevalence of overweight [body mass index [BMI] ≥ 24 kg/m^2^] in adults (>18 years) increased from 22.8 to 30.1%, whereas that of obesity (BMI ≥ 28 kg/m^2^) increased from 7.1 to 11.9 % from 2002 to 2012 (5).

Assisted reproductive technology (ART)-conceived infants tend to be born earlier and at lower birth weights than naturally conceived infants (6–11). Preterm birth (PTB) and low birth weight (LBW) are leading causes of infant mortality as well as other adverse outcomes (including visual and hearing impairments, intellectual and learning disabilities, and behavioral as well as emotional problems with ART-conceived infants) (12, 13).

We know that pre-pregnancy maternal BMI is an important indicator of pregnancy outcomes (14, 15). Obese pregnant women are more likely to have hypertensive disorders of pregnancy, gestational diabetes, post-partum hemorrhage, difficult delivery, macrosomia, and stillbirth (16, 17). However, there is a paucity of data to demonstrate the association between pre-pregnancy maternal BMI and fetal growth for ART-conceived infants (18, 19). Thus, we aimed at evaluating the impact of pre-pregnancy maternal BMI on perinatal outcomes of ART-conceived singletons. We collected and analyzed relevant data from a 10-year registry to compare the gestational age and birth weight among different maternal BMI groups in a single ART center in Xi'an, Shaanxi province, Northwest China.

## Materials and Methods

### Study Design and Population

**Figure 1:**
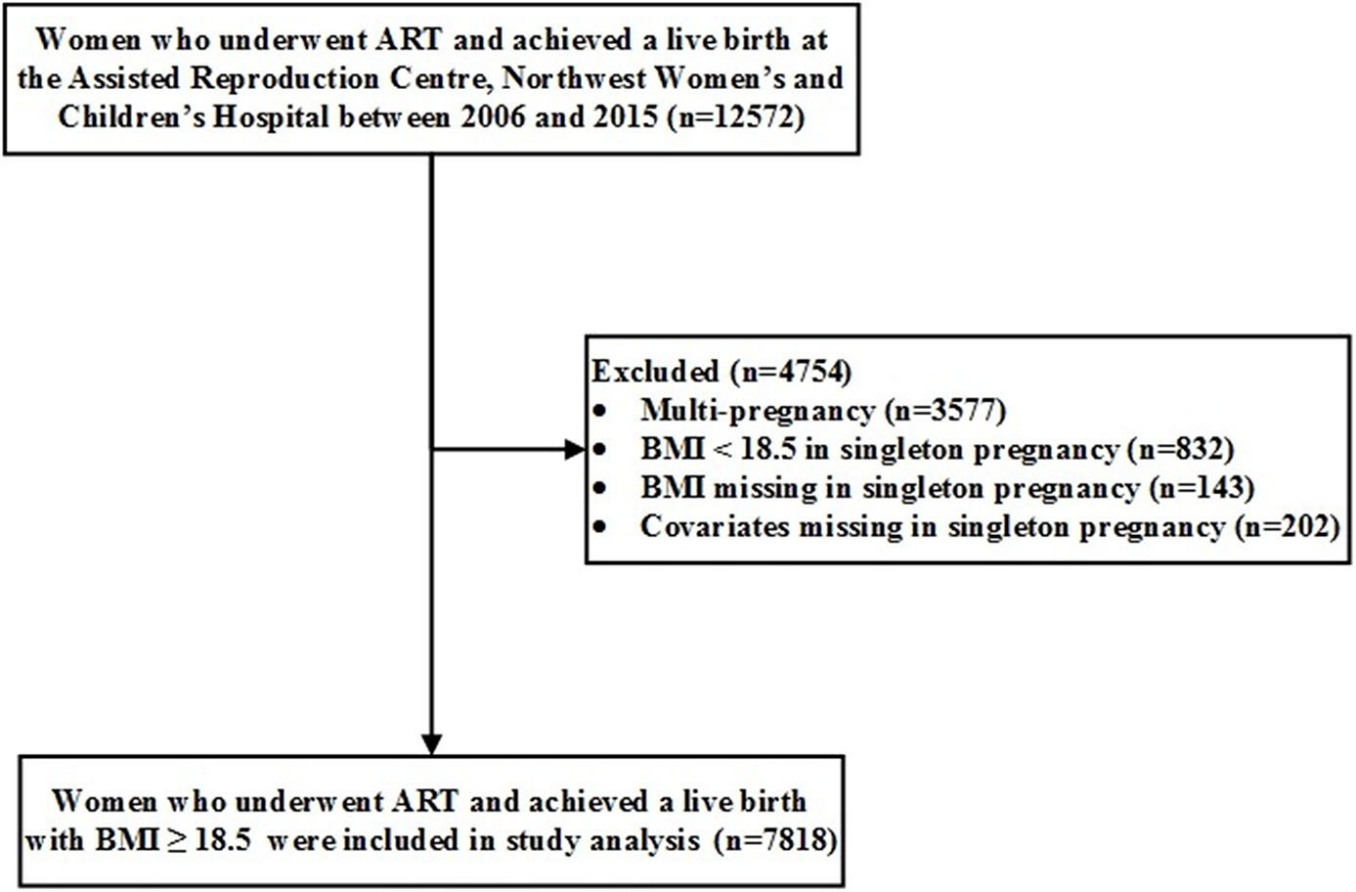
Eligibility assessment with exclusion criteria.

We conducted a retrospective cohort study using the 10-year registry (2006–2015) from a single ART center at Northwest Women's and Children's Hospital, Xi'an, Shaanxi province, Northwest China. We enrolled 12,572 mothers with live deliveries of *in vitro* fertilization (IVF)/intracytoplasmic sperm injection (ICSI) infants. Thereafter, we excluded 3,577 multiple births, 832 mothers with BMI of <18.5 kg/m^2^, 143 mothers with missing pre-pregnancy BMI, and 202 mothers with missing covariates, leaving us with 7,818 mothers with their singletons for data analyses (Figure 1).

We reported all ART birth outcomes (including gestational age, birth weight, and infant sex) in the Shaanxi Assisted Reproduction Database in Shaanxi province of China. In this study, we collected all ART birth outcomes from the Shaanxi Assisted Reproduction Database. Finally, the attending clinician collected and assessed the demographic and ART-treatment data.

### BMI Assessment

The attending nurses measured and recorded the weight and height of participants. We determined BMI as kilograms per square meter using Chinese criteria (20). By this, we separated the participants into three groups. These include the normal-weight group (18.5 kg/m^2^ ≤ BMI <24.00 kg/m^2^), overweight group (24 kg/m^2^ ≤ BMI <28.00 kg/m^2^), and obesity group (BMI ≥ 28 kg/m^2^).

### Definitions of Perinatal Outcomes

The main outcome parameters were gestational age, PTB, birth weight, LBW, macrosomia, small for gestational age (SGA), and large for gestational age (LGA). According to the American College of Obstetricians and Gynecologists (21), gestational age is the number of days from the day of the transfer to birth plus the age of the embryo and 14 days of fertilization. We defined PTB as gestational age <37 weeks (259 days) (22). We measured birth weights using electronic scales (accuracy of 10 g). We defined LBW as birth weight <2,500 g (23). We defined macrosomia as birth weight ≥ 4,000 g (23). We calculated sex- and gestational age-adjusted birth weight z-scores and percentiles according to the International Fetal and Newborn Growth Consortium for the 21st Century (23). We defined SGA as birth weight <10th percentile for that gestational age (21). We defined LGA as birth weight > 90th percentile for that gestational age (23).

### Confounding Variables

Based on the literature (24, 25), we collected all potential correlated factors of perinatal outcomes from the records. These include the year of embryo transfer (2006–2009, 2010–2012, or 2013–2015), maternal age, gravidity (0, 1–2, or, ≥3), parity (0 or ≥1), main etiology of infertility (tubal factor, ovarian factor, male factor, or other reasons), sperm donation (yes or no), fertilization method (ICSI, IVF, or IVF + ICSI), fresh/frozen embryo transfer, blastocyst/cleavage-stage transfer, assisted hatching (yes or no), antral follicle count, basal serum follicle-stimulating hormone (FSH) level, endometrial thickness, maternal smoking history (yes or no), and gestational weight gain.

### Ethical Approval

In January 2018, the Human Research Ethics Committee of the Northwest Women and Children Hospital approved this study and waived the need to obtain informed consent (no. 2018002).

### Statistical Analysis

We expressed continuous variables as mean ± standard deviation, whereas categorical variables as counts and proportions. We performed chi-square tests to compare the categorical variables. Conversely, we performed an analysis of covariance (normally distributed variables) and the Kruskal–Wallis test (abnormally distributed variables) to compare continuous variables among the groups.

We used both univariate and multivariate logistic regression models to analyze the relationship between maternal BMI and PTB, LBW, macrosomia, SGA, and LGA in singleton infants. We adjusted all multivariate analyses for all baseline covariates. Furthermore, we adjusted all baseline covariates and gestational age for LBW and macrosomia. In addition, we demonstrated the effects of maternal BMI on perinatal outcomes in the subgroup of fresh/frozen embryo transfer.

We performed statistical analyses using the SAS software package (version 9.4, SAS Institute Inc., Cary, NC, USA). We considered all *p* < 0.05 as statistically significant.

## Results

### Participants' Characteristics

We recruited 7,817 ART mothers and their singletons. We classified them into normal-weight, overweight, and obese groups according to the classification and evaluation criteria of weight for a Chinese adult. The mean (± SD) age of the participants was 29.97 (± 4.07) years. Furthermore, 5,239 (67.01%) were embryos transferred between 2013 and 2015, and 5,584 (71.42%) were IVF. Table 1 compares the baseline characteristics of participants among BMI groups. Overweight and obese mothers were older and more likely to have larger gravidity and parity, larger antral follicle count, larger endometrial thickness, ovarian factor for infertility, and blastocyst transfer. Finally, overweight and obese mothers were more likely to have lower basal serum FSH levels, lesser sperm donation, and lower gestational weight gain.

**Table 1:** Baseline characteristics of participants by pre-pregnancy maternal BMI in ART pregnancies.

	**Normal weight (*n =* 5,897)**	**Overweight (*n =* 1,613)**	**Obesity (*n =* 308)**	**χ^2^/*F-*value**	***P*-value**
BMI, mean ± SD	21.10 ± 1.47	25.45 ± 1.10	29.88 ± 2.21	4394.363	<0.001^*^
Year of embryo transfer, n (%)
2006–2009	459 (7.78)	88 (5.46)	11 (3.57)		
2010–2012	1,552 (26.32)	402 (24.92)	67 (21.75)	23.797	<0.001
2013–2015	3,886 (65.90)	1,123 (69.62)	230 (74.68)		
Maternal age (year), mean ± SD	29.81 ± 4.01	30.40 ± 4.21	30.67 ± 4.24	33.302	<0.001^*^
Gravidity, n (%)
0	3,397 (57.61)	887 (54.99)	169 (54.87)		
1–2	2,040 (34.59)	572 (35.46)	121 (39.29)	10.528	0.032
≥3	460 (7.80)	154 (9.55)	18 (5.84)		
Parity, n (%)
0	5,379 (91.22)	1,414 (87.66)	276 (89.61)	18.701	<0.001
≥1	518 (8.78)	199 (12.34)	32 (10.39)		
Main etiology of infertility, n (%)
Tubal factor	2,859 (48.48)	819 (50.77)	136 (44.16)	66.176	<0.001
Ovarian factor	221 (3.75)	100 (6.20)	36 (11.69)		
Male factor	1,226 (20.79)	270 (16.74)	58 (18.83)		
Other reasons	1,591 (26.98)	424 (26.29)	78 (25.32)		
Sperm donation, n (%)
Yes	400 (6.78)	77 (4.77)	13 (4.22)	10.991	0.004
No	5,497 (93.22)	1,536 (95.23)	295 (95.78)		
Fertilization method, n (%)
ICSI	1,588 (26.93)	387 (23.99)	78 (25.32)		
IVF	4,170 (70.71)	1,192 (73.90)	222 (72.08)	6.534	0.163
IVF + ICSI	139 (2.36)	34 (2.11)	8 (2.60)		
Timing of embryo transfer, n (%)
Fresh embryo transfer	3,424 (58.06)	944 (58.52)	187 (60.71)	0.903	0.637
Frozen embryo transfer	2,473 (41.94)	669 (41.48)	121 (39.29)		
Day 3 or 5, n (%)
Cleavage-stage transfer	3,774 (64.00)	985 (61.07)	181 (58.77)	7.367	0.025
Blastocyst transfer	2,123 (36.00)	628 (38.93)	127 (41.23)		
Assisted hatching, n (%)
Yes	1,646 (27.91)	471 (29.20)	86 (27.92)	1.048	0.592
No	4,251 (72.09)	1,142 (70.80)	222 (72.08)		
Antral follicle count, mean ± SD	12.86 ± 5.23	13.65 ± 5.74	14.41 ± 6.17	33.882	<0.001^*^
Basal serum FSH level (U/L), mean ± SD	6.86 ± 2.54	6.54 ± 2.14	6.21 ± 1.75	55.424	<0.001^*^
Endometrial thickness (mm), mean ± SD	10.73 ± 2.05	10.86 ± 2.11	11.14 ± 2.01	7.344	0.001
Maternal smoking history, n (%)
Yes	18 (0.31)	7 (0.43)	2 (0.65)	1.471	0.479
No	5,879 (99.69)	1,606 (99.67)	306 (99.35)		
Gestational weight gain (kg), mean ± SD	15.30 ± 1.24	13.32 ± 1.38	13.23 ± 1.87	5753.561	<0.001^*^

*SD, standard deviation; IVF, in vitro fertilization; ICSI, intracytoplasmic sperm injection*.

* *Kruskal–Wallis test*.

### Maternal BMI and Gestational Age

Overall, 0.68, 1.59, and 7.65% of all singleton infants born to participant mothers were GA <32 weeks, GA <34 weeks, and GA <37 weeks, respectively. The proportion of infants with GA <37 weeks varied in accordance with maternal BMI for singletons. Among ART women with normal weight, overweight, and obesity, the percentages whose infants were PTB were 6.90, 9.86, and 10.39% (*P* < 0.001), respectively (Table 2).

**Table 2:** Primary perinatal outcomes by pre-pregnancy maternal BMI in ART singletons.

	**Normal weight (*n =* 5,897)**	**Overweight (*n =* 1,613)**	**Obesity (*n =* 308)**	**χ^2^/*F-*value**	***P*-value**
GA, mean ± SD	39.03 ± 1.63	38.83 ± 1.70	38.68 ± 1.93	34.519	<0.001^*^
GA <32 weeks, n (%)	41 (0.70)	8 (0.50)	4 (1.30)	2.582	0.275
GA <34 weeks, n (%)	84 (1.42)	32 (1.98)	8 (2.60)	4.640	0.098
GA <37 weeks, n (%)	407 (6.90)	159 (9.86)	32 (10.39)	19.072	<0.001
BW, mean ± SD	3292.54 ± 507.70	3360.72 ± 562.43	3375.07 ± 595.71	36.977	<0.001^*^
BW <1,500 g, n (%)	31 (0.53)	10 (0.62)	4 (1.30)	3.126	0.209
BW <2,000 g, n (%)	112 (1.90)	38 (2.36)	7 (2.27)	1.456	0.483
BW <2,500 g, n (%)	280 (4.75)	88 (5.46)	17 (5.52)	1.596	0.450
BW ≥ 4,000 g, n (%)	482 (8.17)	205 (12.71)	44 (14.29)	39.955	<0.001
BW z-score, mean ± SD	0.27 ± 1.01	0.50 ± 1.06	0.59 ± 1.05	90.203	<0.001^*^
BW centile, mean ± SD	57.64 ± 28.18	63.93 ± 27.98	65.71 ± 28.93	40.087	<0.001
SGA, n (%)	460 (7.80)	102 (6.32)	20 (6.49)	4.431	0.109
LGA, n (%)	638 (10.82)	270 (16.74)	64 (20.78)	61.287	<0.001

*GA, gestational age; BW, birth weight; LGA, large for gestational age; SGA, small for gestational age; SD, standard deviation*.

* *Kruskal–Wallis test*.

Table 3 displays the crude and adjusted odds ratios of having a PTB singletons infant. After adjusting for baseline covariates (year of embryo transfer, maternal age, gravidity, parity, maternal smoking history, and gestational weight gain), infants with overweight mothers and obese mothers had 43 and 53% increased risk of PTB, respectively, compared with the normal-weight group [overweight vs. normal weight: odds ratio [OR] = 1.43, 95% CI = 1.18–1.74; obesity vs. normal weight: OR = 1.53, 95% CI: 1.04–2.24]. After adjusting all baseline covariates, infants with overweight mothers and obese mothers had 44 and 53% increased risk of PTB, respectively, compared with the normal-weight group (overweight vs. normal weight: OR = 1.44, 95% CI: 1.18–1.75; obesity vs. normal weight: OR = 1.53, 95% CI: 1.04–2.25). In addition, after adjusting for all baseline covariates, a unit rise in maternal BMI led to a 7% increased risk of PTB (OR = 1.07, 95% CI: 1.04–1.10).

**Table 3:** Effects of pre-pregnancy maternal BMI on primary perinatal outcomes: results from univariate and multivariate logistic regression analyses.

**Birth outcomes**	**Model 1**	**Model 2**	**Model 3**
	**Crude OR (95% CI), *P*-value**	**Adjusted OR (95% CI), *P*-value**	**Adjusted OR (95% CI), *P*-value**
PTB
Normal weight	Ref	Ref	Ref
Overweight	1.48 (1.22, 1.79), <0.001	1.43 (1.18, 1.74), <0.001	1.44 (1.18, 1.75), <0.001
Obesity	1.56 (1.07, 2.29), 0.021	1.53 (1.04, 2.24), 0.031	1.53 (1.04, 2.25), 0.030
PTB
BMI	1.07 (1.04, 1.10), <0.001	1.07 (1.04, 1.10), <0.001	1.07 (1.04, 1.10), <0.001
LBW
Normal weight	Ref	Ref	Ref
Overweight	1.16 (0.91, 1.48), 0.244	0.79 (0.57, 1.10), 0.162^a^	0.83 (0.59, 1.16), 0.271^b^
Obesity	1.17 (0.71, 1.94), 0.537	0.70 (0.35, 1.39), 0.305^a^	0.74 (0.37, 1.50), 0.404^b^
LBW
BMI	1.05 (1.01, 1.09), 0.007	0.97 (0.93, 1.02), 0.278^a^	0.98 (0.93, 1.03), 0.500^b^
Macrosomia
Normal weight	Ref	Ref	Ref
Overweight	1.64 (1.37, 1.95), <0.001	1.81 (1.52, 2.17), <0.001^a^	1.78 (1.48, 2.14), <0.001^b^
Obesity	1.87 (1.34, 2.61), <0.001	2.17 (1.54, 3.06), <0.001^a^	2.16 (1.52, 3.06), <0.001^b^
Macrosomia
BMI	1.10 (1.07, 1.12), <0.001	1.12 (1.09, 1.15) <0.001^a^	1.12 (1.09, 1.15), <0.001^b^
SGA
Normal weight	Ref	Ref	Ref
Overweight	0.80 (0.64, 1.00), 0.046	0.79 (0.63, 0.99), 0.042	0.81 (0.65, 1.01), 0.067
Obesity	0.82 (0.52, 1.30), 0.403	0.81 (0.51, 1.29), 0.373	0.82 (0.51, 1.31), 0.413
SGA
BMI	0.97 (0.94, 1.00), 0.041	0.97 (0.93, 1.00), 0.035	0.97 (0.94, 1.00), 0.056
LGA
Normal weight	Ref	Ref	Ref
Overweight	1.66 (1.42, 1.93), <0.001	1.66 (1.42, 1.94), <0.001	1.63 (1.39, 1.90), <0.001
Obesity	2.16 (1.62, 2.88), <0.001	2.16 (1.62, 2.89), <0.001	2.11 (1.57, 2.83), <0.001
LGA
BMI	1.10 (1.08, 1.13), <0.001	1.10 (1.08, 1.13), <0.001	1.10 (1.08, 1.13), <0.001

*Model 2 adjusted year of embryo transfer, maternal age, gravidity, parity, maternal smoking history, and gestational weight gain*.

a *Model adjusted year of embryo transfer, maternal age, gravidity, parity, maternal smoking history, and gestational weight gain and gestational age*.

*Model 3 adjusted all baseline variates (year of embryo transfer, maternal age, gravidity, parity, main etiology of infertility, sperm donation, fertilization method, fresh/frozen embryo transfer, blastocyst/cleavage-stage transfer, assisted hatching, antral follicle count, basal serum FSH level, endometrial thickness, maternal smoking history, and gestational weight gain)*.

b *Model adjusted all baseline variates and gestational age*.

### Maternal BMI and Birth Weight

Overall, 0.58, 2.01, 4.92, and 9.35% of singleton infants were BW <1,500 g, BW <2,000 g, BW <2,500 g, and BW ≥ 4,000 g, respectively. Among ART women in relation to BMI, the percentages whose infants were LBW were 4.75, 5.46, and 5.52% (*P* = 0.450), respectively. Furthermore, the percentages whose infants were macrosomia were 8.17, 12.71, and 14.29% (*P* <0.001), respectively (Table 2).

Table 3 displays the crude and adjusted OR of having LBW and macrosomia infants. After adjusting for baseline covariates and gestational age, infants with overweight and obese mothers recorded 78 and 116% increased risk of macrosomia compared with the normal-weight group, respectively, (overweight vs. normal weight: OR = 1.78, 95% CI: 1.48–2.14; obesity vs. normal weight: OR = 2.16, 95% CI: 1.52–3.06). Furthermore, after adjusting for all baseline covariates and gestational age, a unit rise in maternal BMI led to a 12% increased risk of macrosomia (OR = 1.12, 95% CI: 1.09–1.15).

After adjusting for all baseline covariates and gestational age, infants with overweight and obese mothers had 17 and 26% decreased risk of LBW compared with the normal-weight group (overweight vs. normal weight: OR = 0.83, 95% CI: 0.59–1.16; obesity vs. normal weight: OR = 0.74, 95% CI: 0.37–1.50). Furthermore, with similar adjustments, a unit rise in maternal BMI led to a 2% increased risk of LBW (OR = 0.98, 95% CI: 0.93–1.03). Unfortunately, all associations were statistically not significant.

### Maternal BMI and Small for Gestational Age and Large for Gestational Age

Overall, we recorded 7.44 and 12.43% of singleton infants were SGA and LGA, respectively. The proportion of SGA and LGA infants varied in accordance with maternal BMI for singletons. Among ART women with normal weight, overweight, and obesity, the percentages with SGA infants were 7.80, 6.32, and 6.49% (*P* = 0.109), respectively. Among ART women with normal weight, overweight, and obesity, the percentages with LGA infants were 10.82, 16.74, and 20.78% (*P* <0.001), respectively (Table 2).

Table 3 shows the crude and adjusted OR of having an SGA and LGA infants. After adjusting for all baseline covariates, infants with overweight and obese mothers had 63 and 111% increased risk of LGA compared with the normal-weight group (overweight vs. normal weight: OR = 1.63, 95% CI: 1.39–1.90; obesity vs. normal weight: OR = 2.11, 95% CI: 1.57–2.83). Furthermore, with similar adjustments, a unit rise in maternal BMI led to a 10% increased risk of LGA (OR = 1.10, 95% CI: 1.08–1.13).

Following similar adjustments, infants with overweight and obese mothers had a 19 and 18% decreased risk of SGA, respectively, compared with the normal-weight group (overweight vs. normal weight: OR = 0.81, 95% CI: 0.65–1.01; obesity vs. normal weight: OR = 0.82, 95% CI: 0.51–1.31). In addition, a unit rise in maternal BMI led to a 3% increased risk of SGA (OR = 0.97, 95% CI: 0.94–1.00). Unfortunately, all associations were statistically not significant.

### Maternal BMI and Other Perinatal Outcomes

Overall, 1.97, 5.68, 4.31, 23.62, 2.60, and 32.23% of mothers experienced spontaneous PTB, medically indicated PTB, pre-eclampsia, gestational diabetes mellitus, post-partum hemorrhage, and cesarean delivery, respectively. In addition, 3.30 and 1.97% of all singleton infants had neonatal respiratory distress syndrome and neonatal intensive care unit recovery, respectively. The proportion of mothers with medically indicated PTB, pre-eclampsia, gestational diabetes mellitus, and cesarean delivery varied in accordance with pre-pregnancy BMI for ART-conceived infants. Among normal-weight, overweight, and obese ART women, the percentages of mothers with medically indicated PTB were 4.95, 7.94, and 7.79% (*P* <0.001), respectively. The percentages of mothers with pre-eclampsia were 3.73, 5.95, and 6.82% (*P* <0.001), respectively. The percentages of mother with gestational diabetes mellitus were 20.14, 32.86, and 41.88% (*P* <0.001), respectively. Finally, the percentages of mothers with cesarean delivery were 35.12, 23.81, and 21.10% (*P* <0.001), respectively (Table 4).

**Table 4:** Secondary perinatal outcomes by pre-pregnancy maternal BMI in ART singletons.

	**Normal weight (*n =* 5,897)**	**Overweight (*n =* 1,613)**	**Obesity (*n =* 308)**	**χ^2^/*F-*value**	***P*-value**
PTB
Spontaneous PTB, n (%)	115 (1.95)	31 (1.92)	8 (2.30)	0.659	0.719
Medically indicated PTB, n (%)	292 (4.95)	128 (7.94)	24 (7.79)	23.724	<0.001
Pre-eclampsia, n (%)	220 (3.73)	96 (5.95)	21 (6.82)	20.034	<0.001
Gestational diabetes mellitus, n (%)	1,188 (20.14)	530 (32.86)	129 (41.88)	172.672	<0.001
Post-partum hemorrhage, n (%)	148 (2.51)	46 (2.85)	9 (2.92)	0.720	0.698
Cesarean delivery, n (%)	2,071 (35.12)	384 (23.81)	65 (21.10)	92.391	<0.001
Neonatal RDS, n (%)	197 (3.34)	51 (3.16)	10 (3.25)	0.130	0.937
NICU recovery, n (%)	115 (1.95)	32 (1.98)	7 (1.94)	0.160	0.923

*PTB, preterm birth; RDS, respiratory distress syndrome; NICU, neonatal intensive care unit*.

### Subgroup Analyses

We performed subgroup analyses between maternal BMI and primary perinatal outcomes by the timing of embryo transfer (fresh/frozen embryo transfer) (Table 5). Table 5 shows that the risk of PTB significantly increased with an increase in maternal BMI in frozen embryo transfer group, but the risk of PTB did not significantly increase with an increase in maternal BMI in fresh embryo transfer group (fresh: OR = 1.03, 95% CI: 0.99–1.08; frozen: OR = 1.09, 95% CI: 1.04–1.15). The interaction test between maternal BMI and the timing of embryo transfer on PTB was statistically significant (*P* = 0.030). Furthermore, increased maternal BMI was associated with higher ORs of macrosomia and LGA in fresh embryo than in the frozen embryo transfer group (macrosomia: fresh: OR = 1.15, 95% CI: 1.11–1.20; frozen: OR = 1.10, 95% CI: 1.06–1.15; LGA: fresh: OR = 1.14, 95% CI: 1.10–1.18; frozen: OR = 1.09, 95% CI: 1.04–1.13). Again, the interaction test between maternal BMI and the timing of embryo transfer on LGA was statistically significant (*P* = 0.030).

**Table 5:** Effects of pre-pregnancy maternal BMI on primary perinatal outcomes: results from multivariate logistic model analyses in subgroups.

	**Fresh embryo transfer**		**Frozen embryo transfer**		***P* for interaction**
	**Crude OR (95% CI), *P*-value**	**Adjusted OR (95% CI), *P*-value**	**Crude OR (95% CI), *P*-value**	**Adjusted OR (95% CI), *P*-value**	
PTB
BMI	1.04 (1.00, 1.08), 0.041	1.03 (0.99, 1.08), 0.188^a^	1.11 (1.07, 1.16), <0.001	1.09 (1.04, 1.15), <0.001^a^	0.030^a^
LBW
BMI	1.02 (0.97, 1.07), 0.494	0.96 (0.89, 1.03), 0.257^b^	1.09 (1.04, 1.15), 0.001	1.01 (0.91, 1.11), 0.902^b^	0.428^b^
Macrosomia
BMI	1.13 (1.09, 1.16), <0.001	1.15 (1.11, 1.20), <0.001^b^	1.07 (1.03, 1.11), 0.001	1.10 (1.06, 1.15), <0.001^b^	0.105^b^
SGA
BMI	0.96 (0.92, 0.99), 0.025	0.95 (0.90, 0.99), 0.016^a^	0.99 (0.93, 1.04), 0.645	0.99 (0.93, 1.06), 0.726^a^	0.334^a^
LGA					
BMI	1.13 (1.10, 1.17), <0.001	1.14 (1.10, 1.18), <0.001^a^	1.08 (1.04, 1.11), <0.001	1.09 (1.04, 1.13), <0.001^a^	0.030^a^

a *Model adjusted all baseline variates (year of embryo transfer, maternal age, gravidity, parity, main etiology of infertility, sperm donation, fertilization method, fresh/frozen embryo transfer, blastocyst/cleavage-stage transfer, assisted hatching, antral follicle count, basal serum FSH level, endometrial thickness, maternal smoking history, and gestational weight gain)*.

b *Model adjusted all baseline variates and gestational age*.

## Discussion

In a large cohort of pregnant women with ART treatment follow-up for perinatal outcomes, we used the Chinese criteria for overweight and obesity instead of the World Health Organization standard for classification. We found that maternal overweight and obesity before pregnancy were significantly associated with a higher risk of PTB, macrosomia, and LGA in singletons. Furthermore, we found significant interactions between maternal BMI and the timing of embryo transfer (fresh/frozen embryo transfer) on PTB and LGA. Fresh embryo transfer increased the effects of maternal BMI on LGA, and frozen embryo transfer increased the effect of maternal BMI on PTB.

In spontaneous pregnancies, maternal overweight or obesity before pregnancy are known risk factors for pregnancy complications and adverse perinatal outcomes (26–28). Previous studies of the effect of overweight or obesity in pregnancies resulting from IVF have shown concern with oocyte numbers and quality and with conception, live birth, and miscarriage (29–32). Therefore, few studies have demonstrated the relationships between overweight/obesity and perinatal outcomes resulting from ART treatment. In our study, we found that singletons born to overweight and obese mothers had 44 and 53% increased risks of PTB, respectively, compared with those born to normal weight after ART treatment. Our findings are in line with previous studies. Dickey et al. (18) found that overweight and obesity were associated with increased risk of PTB in singletons conceived by ART (overweight vs. normal weight: RR = 1.2, 95% CI: 1.2–1.3; Obesity vs. normal weight: RR = 1.4, 95% CI: 1.3–1.5). Kawwas et al. (33) used the national ART Surveillance System including all fresh autologous IVF cycles in the United States from 2008 to 2013 (*n* = 180,855 pregnancies) to find out that obese mothers were associated with increased risk of PTB in singletons (RR = 1.42, 95% CI: 0.36–1.48).

In our study, we also found that maternal overweight and obesity were associated with a higher risk of macrosomia and LGA in ART-conceived singletons. Especially for obese mothers, the risks of macrosomia and LGA in their infants exceeded two times those born to normal-weight mothers. Those associations were similar in spontaneous pregnancies (34–36). Yu et al. (37) conducted a systematic review and meta-analyses that included 37 studies. They reported that pre-pregnancy maternal overweight/obesity increased the risk of having infants with macrosomia (birth weight > 4,000 g) (overweight: OR = 1.53; 95% CI: 1.44–1.63; obesity: OR = 2.00, 95% CI: 1.84–2.18), and LGA (overweight OR = 1.53, 95% CI: 1.44–1.63; obesity: OR = 2.08, 95% CI: 1.95–2.23) (37). Another systematic review and meta-analysis also revealed that maternal obesity is associated to fetal overgrowth (birth weight ≥ 4,000 g: OR = 2.17, 95% CI: 1.92–2.45; birth weight ≥ 4,500 g: OR = 2.77, 95% CI: 2.22–3.45; and LGA: OR = 2.42, 95% CI: 2.16–2.72) (38). Finally, a retrospective cohort study of over 12,000 live-born singleton pregnancies found that infants born to overweight and obese mothers were at higher risk of LGA (overweight: 12.3 vs. 10.5%, *P* = 0.01; obesity: 16.8 vs. 10.5%, *P* <0.001) compared with normal-weight mothers (39).

The mechanism of pre-pregnancy maternal anthropometric parameters affecting neonatal birth weight is unclear. Possible explanations reveal that fetal growth (overweight/obesity) is related to insulin resistance and genetic factors such as insulin-like growth factor—II hypomethylation (40, 41). Pre-pregnancy overweight and obesity are strong predictors of gestational diabetes (42) and are shown to be associated with risks of macrosomia and LGA. We recommend intensification of management strategies to stabilize anthropometric parameters during pre-pregnancy to achieve normal BMI before pregnancy and maintain a proper weight gain during pregnancy.

Wei and collaborators conducted a multicenter, non-blinded, randomized control trial to compare pregnancy outcomes, and obstetrical/perinatal complications after fresh/frozen single blastocyst transfer in ovulating women. They found that frozen single blastocyst transfer was associated with higher birth weight in singletons (frozen vs. fresh: 3,407.9 vs. 3,293.1 g, *P* = 0.0018) and higher risks of LGA (frozen vs. fresh: 18.6 vs. 11.6%, *P* = 0.0067) (43). Furthermore, we investigated the impact of pre-pregnancy maternal underweight on birth outcomes among ART-conceived singletons in a previous retrospective cohort. An interaction was found between maternal underweight and timing of embryo transfer (fresh/frozen embryo transfer) on gestational age (underweight vs. normal weight: fresh: difference = −0.07 week, 95% CI: −0.22 to 0.09 week; frozen: difference = 0.15 week, 95% CI: −0.05 to 0.36 week; *P* for interactio*n* = 0.038) (25). In our study, we found a significant interaction between pre-pregnancy maternal BMI and timing of embryo transfer (fresh/frozen embryo transfer) on PTB and LGA in ART-conceived singletons. Fresh embryo transfer increased the effects of pre-pregnancy maternal BMI on LGA. Meanwhile, frozen embryo transfers increased the effect of pre-pregnancy maternal BMI on PTB. Our study added evidence of the interaction between maternal BMI and timing of embryo transfer on perinatal outcomes of ART-conceived singletons. The results of our study are a clue that more focus should be on PTB in ART women with overweight or obesity and with frozen embryo transfer, whereas more focus on LGA in ART women with overweight or obesity and with fresh embryo transfer.

In contrast to other large studies of the relationship of BMI to perinatal outcomes (18, 19, 33), we could demonstrate the interactions between pre-pregnancy maternal BMI and timing of embryo transfer (fresh/frozen embryo transfer) on PTB and LGA in ART-conceived singletons. In addition, we were able to define the time of conception and birth with exactitude and achieve accurate birth weight from clinical records. Again, we could collect ART treatment procedures in all participants and make suitable adjustments for models of perinatal outcomes. Furthermore, we classified overweight and obesity according to Chinese standards, more suited for our population. Nevertheless, just like any study, we encountered some limitations. Firstly, although we used multivariable regressions to control for potential confounders among the groups, unmeasured covariates could confound the study because of limited databases. Furthermore, the Chinese criteria for BMI were used instead of the World Health Organization standard for classification in this study and should be considered when discussed out of the Chinese scope.

## Conclusion

In conclusion, our findings indicate that pre-pregnancy maternal overweight and obesity were associated with increased risks of PTB, macrosomia, and LGA. Furthermore, we could demonstrate the interactions between maternal BMI and the timing of embryo transfer (fresh/frozen embryo transfer) on PTB and LGA. Our findings were important for the prevention of adverse perinatal outcomes in ART treatment. Women before ART should maintain a normal BMI for the prevention of adverse perinatal outcomes.

## Data Availability Statement

The raw data supporting the conclusions of this article will be made available by the authors, without undue reservation.

## Ethics Statement

The studies involving human participants were reviewed and approved by The Human Research Ethics Committee of the Northwest Women's and Children's Hospital. Written informed consent for participation was not required for this study in accordance with the national legislation and the institutional requirements.

## Author Contributions

PQ, SD, WS, and JS conceived and designed the study. PQ, DZ, YM, SD, WS, and JS drafted and revised the manuscript. PQ and DZ analyzed and interpreted the data. PQ and MW collected and cleared the data. All authors have read and approved the final version of the manuscript.

## Conflict of Interest

The authors declare that the research was conducted in the absence of any commercial or financial relationships that could be construed as a potential conflict of interest.
